# Muskelrelaxierung 2026 – 50 Jahre Suche nach einem idealen Relaxans

**DOI:** 10.1007/s00101-026-01707-w

**Published:** 2026-06-19

**Authors:** Thomas Krönauer, Felix Girrbach, Philipp Simon, Axel R. Heller

**Affiliations:** Anästhesiologie und Operative Intensivmedizin, Medizinische Fakultät, Universität Augsburg, Universitätsklinikum Augsburg, Stenglinstr. 2, 86156 Augsburg, Deutschland

**Keywords:** Rocuronium, Sugammadex, Succinylcholin, Neostigmin, Neuromuskuläres Monitoring, Rocuronium, Sugammadex, Suxamethonium, Neostigmin, Neuromuscular monitoring

## Abstract

Muskelrelaxierung ist aufgrund des anschaulichen Zusammenspiels zwischen Physiologie und Pharmakologie und den unmittelbar einsetzenden Effekten seit seiner klinischen Einführung eines der spezifischsten Themengebiete der Anästhesiologie.

Über die klassische anästhesiologische Indikation bei Atemwegssicherung hinaus wurden zahlreiche moderne Operationsverfahren und Eingriffe erst durch die Weiterentwicklung der Muskelrelaxierung ermöglicht. Aber auch vor den damit einhergehenden Gefahren und Komplikationen wird seit Jahrzehnten gewarnt.

Im Zuge des Auslaufens des Patentschutzes für Sugammadex im Sommer 2023 schien durch die spezifische Wirkung bei günstigem Nebenwirkungsprofil eine entscheidende Vereinfachung im Management der Muskelrelaxierung erreicht worden zu sein. Knapp zwei Jahre später müssen wir anmerken, dass aus dieser vorteilhaften Entwicklung trotzdem keine vollumfängliche Sicherheit abgeleitet werden kann, sondern im Zusammenhang mit Muskelrelaxierung weiterhin klinische Risiken bestehen.

Deshalb sind in diesem Bereich fundierte physiologische und pharmakologische Kenntnisse ebenso wichtig wie ein konsequent sorgfältiges Arbeiten anhand der aktuellen Vorgaben der anästhesiologischen Fachgesellschaften.

## Abstract (en)

Since its clinical introduction, muscle relaxation has been one of the most specific topics in anesthesiology, due to the clear interaction between physiology and pharmacology and the immediately apparent effects.

Beyond the classical indications in anesthesia for securing the airway, many modern surgical procedures and interventions would not be possible without the development of muscle relaxation; however, warnings about the associated risks and complications have also been issued for decades.

Following the expiration of the patent protection of sugammadex in summer 2023, its specific effects and favorable side-effect profile have made a significant contribution to the simplification of muscle relaxation management; however, nearly 2 years later it is important to acknowledge that this advantageous development does not guarantee complete safety and that clinical risks continue to exist in connection with muscle relaxation.

Therefore, in this field profound physiological and pharmacological knowledge stay just as important as a consistent and thorough work ethic in accordance with the current guidelines of the anesthesia professional associations.

## Geschichte

Die indigene Bevölkerung Südamerikas verwendete zur Jagd ein aus verschiedenen Pflanzen gewonnenes Pfeilgift, das bereits im 16. Jahrhundert im Rahmen der europäischen Eroberungsexpeditionen beschrieben wurde. *Curare* ist dabei ein Sammelbegriff für verschiedene Substanzen, dessen Herstellung auch von Alexander von Humboldt auf seiner Reise zum Orinoco in seinen Reisebericht aufgenommen wurde. Nach verschiedenen Tierexperimenten im 19. Jahrhundert durch Claude Bernard und Jakob Pal verwendete Arthur Läwen das Derivat Curarin als einer der ersten Ärzte am Menschen, um den Verschluss der Bauchdecke durch die Entspannung der Muskulatur zu erleichtern. Die Beschreibung wurde 1912 in *Beiträge zur klinischen Chirurgie* publiziert [44]. Leider geriet diese Erkenntnis mutmaßlich im Zusammenhang mit dem Beginn des 1. Weltkriegs und den resultierenden Folgen für längere Zeit in Vergessenheit. Als klinische Einführung der Muskelrelaxierung gilt heutzutage die Publikation von Griffith und Johnson aus dem Jahr 1942 [31].

Im Zusammenhang mit der Entwicklung des Fachgebiets Anästhesiologie wurde die Bedeutung der Muskelrelaxanzien schnell erkannt. In den 1970er-Jahren formulierte Savarese bereits die Anforderungen an das ideale Muskelrelaxans [65]. Ebenso wurde bereits vor über 40 Jahren die Bedeutung des neuromuskulären Monitorings (NMM) proklamiert, und einige der bis heute vorherrschenden Schwierigkeiten der Messungenauigkeit und Interpretation wurden beschrieben [2, 77].

Aufgrund dieser weiterhin existierenden Herausforderungen aus dem Themenkomplex „Muskelrelaxierung und NMM“ wurden in den vergangenen Jahren verschiedene Empfehlungen und Leitlinien diverser anästhesiologischer Fachgesellschaften veröffentlicht, deren wesentliche Aussagen sich inhaltlich stark decken und auf die im Folgenden eingegangen werden soll [22, 42, 54, 74].

## Gibt es das ideale Muskelrelaxans und die ideale Substanz zur Antagonisierung?

Im Zusammenhang mit der zunehmenden Bedeutung der Muskelrelaxierung wurde bereits 1975 von Savarese formuliert, welche Eigenschaften das „ideale“ Muskelrelaxans haben sollte [65]. Sowohl ein schneller Wirkeintritt als auch eine kurze Wirkdauer mit zügiger Erholung waren neben einer geringen Gefahr pharmakologisch aktiver Metaboliten und unerwünschter Nebenwirkungen die Kernforderungen.

Diese „idealen Eigenschaften“ werden bis heute von keiner Einzelsubstanz erfüllt, durch Kenntnis der spezifischen pharmakologischen Charakteristika sind aber – je nach Indikation und Eingriff – alle gängigen Substanzen aus Tab. 1 sinnvoll einsetzbar. Im Rahmen der Weiterbildung in Anästhesiologie muss es (nicht nur für Muskelrelaxanzien) das Ziel sein, den Umgang mit verschiedenen Substanzen zu erlernen, um auch bei Vorliegen spezifischer Kontraindikationen oder bei Nichtverfügbarkeit einzelner Substanzen alternative Medikamente sicher anwenden zu können.

Die Kombination aus Rocuronium und Sugammadex kommt den Wünschen von 1975 im Jahr 2026 am nächsten

Die Kombination aus Rocuronium und Sugammadex kommt den vor 50 Jahren formulierten Wünschen im Jahr 2026 am nächsten. Die beiden Ziele *vollständige neuromuskuläre Erholung vor Extubation* und *an die chirurgischen Bedürfnisse angepasste Relaxierungstiefe* während des Eingriffs lassen sich bei adäquater Anwendung mit dieser Kombination vermutlich mit den geringsten Nebenwirkungen erreichen, sind aber natürlich auch mit anderen Substanzen möglich. Seit der Einführung von Sugammadex ist es nachweislich zu einer Steigerung der verwendeten Rocuroniumdosen gekommen [79]. Auch nach Reversierung ist allerdings immer ein quantitatives NMM anzuwenden, um Restblockaden und Komplikationen zu vermeiden. Nachdem die Effizienz für Sugammadex als in diesem Kontext erste enkapsulierende Substanz gezeigt werden konnte [55, 56], wurden mittlerweile ebenfalls Anforderungen an eine ideale Substanz zur Antagonisierung bzw. zur Reversierung neuromuskulärer Blockaden beschrieben [35]. Es wird auch weiterhin an neuen Substanzen geforscht, mit dem Ziel einer fortschreitenden Verbesserung [37, 83].

**Tab. 1:** Gängige Muskelrelaxanzien in der klinischen Verwendung

Name und Substanzgruppe	Dosis zur Intubation [mg/kgKG]	Klinische Einführung
*Depolarisierend*		
Succinylcholin = Suxamethonium	1,0	1951
*Nichtdepolarisierend*		
Benzylisochinoline		
Atracurium	0,5	1980
Cis-Atracurium	0,1	1995
Mivacurium	0,2	1997
Aminosteroide		
Pancuronium	0,1–0,15	1960
Vecuronium	0,1–0,15	1980
Rocuronium	0,6 (RSI: 0,9–1,2)	1992

*RSI* Rapid Sequence Induction, *KG* Körpergewicht

## Die anhaltende Diskussion um Succinylcholin

Das älteste und einzige depolarisierende Muskelrelaxans Succinylcholin wird aufgrund seiner Nebenwirkungen und den verfügbaren Alternativen außerhalb von Notfällen nur noch selten verabreicht [5, 83]. Seine Verwendung beschränkt sich auf die Notfallintubation im Rahmen einer RSI in der prähospitalen Notfallmedizin oder bei innerklinischen Intubationen mit erhöhtem Aspirationsrisiko wie beispielsweise aufgrund eines Dünndarmileus oder (Notfall‑)Kaiserschnitts, wobei bei diesen Indikationen zunehmend häufiger Rocuronium Anwendung findet [71]. Insbesondere in Notfallsituationen bietet Succinylcholin im Rahmen einer RSI jedoch weiterhin die Vorteile des schnellsten Wirkeintritts [75], eines höheren Anteils an exzellenten/akzeptablen Intubationsbedingungen [75] und einer höheren Erfolgsrate des ersten außerklinischen Intubationsversuchs [33], hat jedoch aufgrund zahlreicher Kontraindikationen auch Nachteile. Nach einer Nutzen-Risiko-Abwägung und unter strenger Beachtung der Kontraindikationen sowie Kenntnis der Therapie etwaiger Nebenwirkungen ist Succinylcholin bei diesen Fällen somit weiterhin eine Option, für die mit Rocuronium (ggf. + Sugammadex) eine adäquate Alternative existiert [22]. Die Studienlage zu Gleichwertigkeit liefert unterschiedliche Ergebnisse zwischen marginal ausfallenden, nichtsignifikanten Unterschieden [57] und scheitert an der „Non-inferiority“ – Hürde [33].

Bei Notfallintubationen ist Succinylcholin unter Beachtung der Kontraindikationen weiterhin eine Option

Bei elektiven Eingriffen ohne erhöhtes Aspirationsrisiko im Jahr 2026 ist die Verwendung von Succinylcholin allerdings gemäß der vorherrschenden Meinung aus der Literatur mit Ausnahme einiger Spezialfälle (z. B. Elektrokrampftherapie) als obsolet zu bezeichnen. Ein weiterer bislang wenig beachteter Aspekt ist, dass sich auch nach Gabe von Succinylcholin das Risiko für postoperative pulmonale Komplikationen erhöht [67] und folglich ein adaptiertes neuromuskuläres Monitoring angezeigt ist [68]. Zusammengefasst gibt es im Jahr 2026 weiterhin ein Für und Wider in der Diskussion um Alternativen zu Succinylcholin im Rahmen der RSI [30, 69], und in verschiedenen Publikationen wird trotz der kontroversen Diskussion aktuell keine vollständige Abkehr von der Substanz empfohlen [5, 23].

## Antagonisierung und Reversierung

Bei der Antagonisierung von Muskelrelaxanzien mit einem Acetylcholinesterasehemmer, z. B. Neostigmin, handelt es sich um eine indirekte Aufhebung der kompetitiven Hemmung des nikotinergen Acetylcholinrezeptors durch Erhöhung der Acetylcholinkonzentration im synaptischen Spalt. Im Gegensatz dazu vermittelt Sugammadex seine reversierende Wirkung über direkte kovalente Bindung und Enkapsulierung von steroidalen Muskelrelaxanzien (Rocuronium > Vecuronium). Durch das resultierende Konzentrationsgefälle diffundiert das Relaxans von seinem Wirkort im synaptischen Spalt zurück in das Kompartiment Blut, wodurch die schnelle Wirkentfaltung erklärt werden kann. Zur Verringerung der über muskarinerge Acetylcholinrezeptoren vermittelte Nebenwirkungen sollte Neostigmin mit Glykopyrronium (wenn nicht verfügbar Atropin) kombiniert werden. Die Vorteile der Verwendung von Glykopyrronium bestehen in der günstigeren Pharmakokinetik [3] und dem Fehlen zentraler anticholinerger Nebenwirkungen, da Glykopyrronium als quartäre Ammoniumverbindung im Gegensatz zu Atropin die Blut-Hirn-Schranke nur eingeschränkt passieren kann. Eine Antagonisierung mit Neostigmin ist aufgrund des Ceiling-Effekts [4] erst ab einem oberflächlichen Block (TOF-Quotient > 0,2) [22] bzw. minimalem Block (TOF-Quotient > 0,4) [74] sinnvoll.

Die Überlegenheit von Sugammadex gegenüber Neostigmin konnte bei vergleichbarer Sicherheit der Anwendung [63] bzgl. der Dauer bis zum Wirkeintritt [38] und der Effektivität [8] nachgewiesen werden, wobei die häufigsten Nebenwirkungen Bradykardie und Bronchospasmus in der überwiegenden Mehrheit vorrübergehend und von geringem Schweregrad der Ausprägung verlaufen sind [63].

## Postoperative Restblockade

Eriksson konnte bereits in den 1990er-Jahren zeigen, dass selbst bei geringster Relaxierungstiefe (TOF-Quotient: 0,6–0,9) bereits Schluckbeschwerden auftreten [19] und vor allem die unabhängig von der motorischen Endplatte über Chemorezeptoren vermittelte Atemantwort auf Hypoxie durch die Gabe von Muskelrelaxanzien beeinträchtigt wird [18]. Dies kann im perioperativen Kontext durch die Nebenwirkungen von Opioiden oder Sedativa weiter aggraviert werden und erhöht zusätzlich das Risiko einer Hypoxie.

Bei einer TOF-Ratio >  0,9 vor Extubation ist die pulmonale Komplikationsrate höher als bei >  0,95

Bezogen auf die Inzidenz postoperativer pulmonaler Komplikationen in Abhängigkeit einer postoperativen Restblockade („residual neuromuscular blockade“, RNMB) konnte sogar eine signifikanter Unterschied zwischen einer TOF-Ratio > 0,9 und > 0,95 vor Extubation nachgewiesen werden [6]. Die neuromuskuläre Erholung sollte also möglichst vollständig sein.

## Welche Gründe tragen zu Restblockaden im Aufwachraum bei, und welche Erkenntnisse wurden offenbar bislang noch nicht breit genug kommuniziert?

Eine alleinige klinische Beurteilung der neuromuskulären Erholung wird in den Guidelines der American Society of Anesthesiology (ASA) explizit abgelehnt und aufgrund der oben aufgeführten Problematik wird ein quantitatives gegenüber einem qualitativen NMM bevorzugt [74]. Trotz der eben dargestellten Erkenntnisse und der Tatsache, dass die Wirkdauer eines Muskelrelaxans auch bei einmaliger Gabe vor allem von patientenindividuellen und nicht von zeitabhängigen Faktoren abhängt [11], wird die Entscheidung zur Extubation oftmals weiterhin anhand von klinischen Kriterien wie Atemzugvolumen oder der Dauer seit der letzten Gabe eines Muskelrelaxans abhängig gemacht. In einer Umfrage wurde die Problematik von Restblockaden eher bei „anderen“ Anästhesistinnen und Anästhesisten (60 %) gesehen als bei sich „selbst“ (16 %) [78].

## Recurarisierung 2026

Im Gegensatz zu den „klassischen“ Fällen der Recurarisierung bei Verwendung von Acetylcholinesterasehemmern, die mit einem Wiedereinsetzen der Relaxanswirkung durch kompetitive Hemmung der Acetylcholinrezeptoren aufgrund unterschiedlicher Halbwertszeiten der Substanzen erklärt wurde, bindet Sugammadex Rocuronium kovalent und eliminiert es dadurch dauerhaft. Bei längerer Verwendung und hohen Dosen von Rocuronium kann es allerdings zu einer Anreicherung von Rocuronium im peripheren Kompartiment kommen.

Ein Rebound nach Rocuronium- und Sugammadex-Gabe bei bereits extubierten Patienten mit einem rezidivierenden neuromuskulären Block lässt sich eventuell durch die Umverteilung von ungebundenem Rocuronium aus dem peripheren in das zentrale und effektive Kompartiment erklären, nachdem Sugammadex bereits ausgeschieden worden ist (Infobox 1). Die Clearance aus dem peripheren Kompartiment nach lang andauernder Rocuroniumgabe beträgt 0,03 l/min [81] gegenüber einer Sugammadex-Blut-Clearence von 0,12 l/min [29]. Zu den Risikofaktoren gehören neben hohen Rocuroniumdosen die kontinuierliche Verabreichung und/oder die längere Aufrechterhaltung eines tiefen neuromuskulären Blocks sowie eine zu niedrige Sugammadexdosierung [36].

Das stöchiometrische Verhältnis zwischen Sugammadex und Rocuronium beträgt 3,6:1 [7], somit ist theoretisch zur Reversierung von Rocuronium ungefähr die vierfache Dosis an Sugammadex erforderlich, wenn Metabolisierung und Exkretion unbetrachtet bleiben. Die erforderliche Dosis von Sugammadex wird unabhängig davon anhand der Tiefe des neuromuskulären Blocks ermittelt. Es ist legitim, die Dosierung von Sugammadex anhand des tatsächlichen Körpergewichts zu berechnen, während die Dosierung von Muskelrelaxanzien bezogen auf eine vereinfachte Annäherung an das Idealgewicht (Körpergröße in cm – 100) erfolgen sollte. Dieses Vorgehen kann darüber hinaus den Wirkeintritt der Reversierung beschleunigen [34].

### Infobox 1 Theoretisch-pharmakologische Überlegungen

**Abb. 1:**
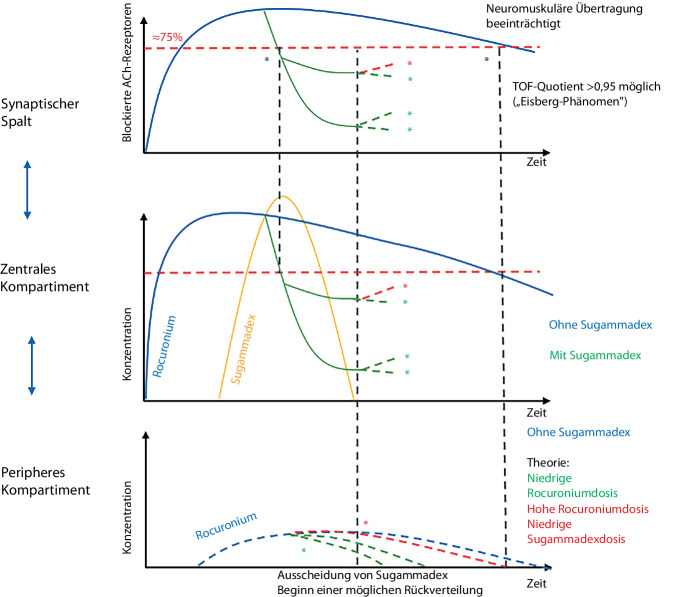
Pharmakokinetik und Pharmakodynamik von Rocuronium und Sugammadex (stark vereinfachte schematisch-theoretische Überlegung). Gestrichelte Linie Schwarz mit *: Extubation gemäß neuromuskulärem Monitoring möglich. Gestrichelte Linie Rot mit *: Recurarisierung möglich aufgrund von Umverteilung aus dem peripheren Kompartiment nach Eliminierung von Sugammadex und noch besetzten Rezeptoren im synaptischen Spalt. Gestrichelte Linie Grün mit *: Recurarisierung unwahrscheinlich aufgrund fehlender Umverteilung oder Eliminierung eines ausreichenden Anteils von Rocuronium vor Beginn der Umverteilung. © mod. nach Krönauer et al.: Clinically significant recurarisation in the postanaesthesia care unit (…) [43]

Nach der Verabreichung hoher Dosen von Rocuronium könnte die gewählte Dosis Sugammadex vorübergehend ein für die Extubation ausreichendes TOF-Verhältnis > 95 % erreichen, aber gemäß dem „Eisberg-Phänomen“ könnten weiterhin bis zu 75 % der Rezeptoren blockiert sein (Abb. 1). In dieser Situation würde eine geringe Menge an Rocuroniummolekülen, die nach Elimination von Sugammadex aus dem peripheren Kompartiment rückverteilt wird, möglicherweise ausreichen, um einen höheren Grad der neuromuskulären Blockade wiederherzustellen [15, 43]. Klinische oder experimentelle Evidenz zur Bestätigung dieser Theorie ist jedoch bisher nicht verfügbar.

## Besondere Aspekte und Patientinnen- und Patientengruppen

### Frauen unter hormoneller Kontrazeption

Bei Frauen in gebärfähigem Alter kann es möglicherweise bei Gabe von Sugammadex zu einer Wirkabschwächung einer hormonellen Kontrazeption kommen, weshalb aus rechtlichen Gründen eine Aufklärung zur Verwendung einer nichthormonellen Verhütungsmethode nach Gabe von Sugammadex notwendig wird. Die Gabe soll deshalb nur nach entsprechender Indikationsstellung und einer Nutzen-Risiko-Abwägung erfolgen [60], auch wenn die hormonellen Veränderungen nicht so ausgeprägt zu sein scheinen, dass sich daraus schwerwiegende Bedenken bzgl. einer fehlenden Empfängnisverhütung ergeben [12, 13]. Bei dieser Patientinnengruppe könnte primär ein anderes Muskelrelaxans (z. B. (Cis‑)Atracurium) verwendet werden. Als Alternativen bieten sich der Verzicht auf eine Reversierung mit Sugammadex oder eine Antagonisierung mit Neostigmin an, die auch bei Verwendung von Rocuronium ab einem TOF-Quotient > 0,2 möglich ist.

### Schwangerschaft und Stillzeit

Die Inzidenz eines unerwartet schwierigen Atemwegs ist bei schwangeren Patientinnen im Gegensatz zu elektiven Eingriffen erhöht [41, 58], vor allem im Rahmen einer RSI zur Not-Sectio caesarea [10]. Aufgrund des ebenfalls erhöhten Aspirationsrisiko wird bereits während der Schwangerschaft häufiger eine RSI durchgeführt, welche oft ab dem zweiten, spätestens ab dem dritten Trimenon empfohlen wird [17]. Deshalb kommt es regelhaft auch bei kurzen Eingriffen zum Einsatz von Rocuronium in RSI-Dosierung und unvollständiger Spontanerholung am Ende des Eingriffs. Sugammadex kann durch die Bindung von Progesteron und einer potenziellen Reduktion der Plasmaspiegel möglicherweise einen Einfluss auf verschiedene Vorgänge der Früh- und Spätschwangerschaft sowie am Übergang zur Stillperiode ausüben. Es fehlt diesbezüglich u. a. aufgrund der ethischen Schwierigkeiten bei Medikamentenstudien während der Schwangerschaft zu mehreren Fragestellungen an ausreichender Evidenz. Die Society for Obstetric Anaesthesia and Perinatology hat sich daher im Jahr 2019 gegen die Anwendung von Sugammadex in der Frühschwangerschaft und für Vorsicht bei Gaben zu späteren Schwangerschaftszeitpunkten ausgesprochen [80]. Es häufen sich gleichzeitig Berichte und Fallserien zur Anwendung von Sugammadex bei Schwangeren ohne negative Folgen auf den Schwangerschaftsverlauf, sodass die Empfehlung gegen die Anwendung von Sugammadex bei Schwangeren kontrovers diskutiert wird, es aber nichtsdestotrotz bei einer Nutzen-Risiko-Abwägung bleibt [26, 60, 80].

Analog ist die Debatte zum Vorgehen bei Sectio caesarea in Intubationsnarkose wegen geringer Evidenz noch nicht abgeschlossen, und es ist zu früh, um eine Empfehlung zur Verwendung von Succinylcholin oder Rocuronium/Sugammadex abzugeben. Eine Verwendung von Sugammadex nach Beginn der Stillzeit erscheint unproblematisch.

### Kinder

Lange Zeit wurde proklamiert, dass Sugammadex bei Kindern unter 2 Jahren als Off-label-Verwendung unter besonderer Vorsicht sicher möglich sei [32]. Kürzlich wurde Sugammadex sowohl durch die European Medicines Agency (EMA) [20] als auch die Food and Drug Administration (FDA) [21] ohne Altersbeschränkung freigegeben. Es konnte darüber hinaus gezeigt werden, dass Dosierungen von 2 und 4 mg/kgKG auch bei Kinder unter 2 Jahren ohne schwerwiegende Nebenwirkungen gut toleriert wurden [47] und bezogen auf das Auftreten von PONV Vorteile gegenüber Neostigmin vorliegen könnten [9]. Da die ED95 bei kleineren Kindern 0,25 mg/kgKG beträgt, sollte allerdings die Rocuroniumdosis zur Intubation auf 0,5 mg/kgKG begrenzt werden [73]. Mittlerweile ist mit wenigen Ausnahmen bei Spezialfällen eine Zwischenbeatmung auch im Rahmen der RSI von Kindern obligat, und da dabei – im Gegensatz zur verwendeten Bezeichnung „rapid“ – die Geschwindigkeit der Intubation gegenüber der Oxygenierung sowie adäquater Tiefe von Narkose und Muskelrelaxierung eine nachrangige Priorität einnimmt, sollte bei der Dosierung der Muskelrelaxanzien eine unnötig hohe Dosis vermieden und ein nichtdepolarisierendes Relaxans verwendet werden. Ein neuromuskuläres Monitoring durch EMG kann in dieser Patientengruppe valide Ergebnisse liefern, wohingegen die AMG-Messung eine Herausforderung darstellt [76]. Insbesondere bei Neugeborenen und Säuglingen oder nach Reversierung aus tiefen neuromuskulären Blockaden muss in der postoperativen Phase eine besondere Wachsamkeit auf Symptome einer möglichen Recurarisierung gelegt werden [64, 76].

### Geriatrische Patientinnen und Patienten

Ältere Patientinnen und Patienten haben aufgrund verschiedener Ursachen (v. a. Veränderung der Organfunktionen sowie Änderung von Muskelmasse und Verteilungsvolumen) ein erhöhtes Risiko für eine postoperative neuromuskuläre Restblockade [24, 49] und bedürfen besonderer Wachsamkeit beim NMM.

### Patientinnen und Patienten auf der Intensivstation

Auch in der Intensivmedizin ist der Einsatz von Muskelrelaxanzien zur endotrachealen Intubation obligat, und es sollte sich zur Vermeidung einer Awareness nach der Induktion eine adäquate Sedierung anschließen. Dabei sollten vergleichbare Aspekte wie im OP beachtet werden, wobei durch Veränderungen an Organen, Muskulatur und motorischer Endplatte sowohl Wirkeintritt als auch Wirkdauer verändert sein können. Bei einer geplanten Extubation im zeitlichen Zusammenhang mit der Gabe von Muskelrelaxanzien oder direkt nach operativen Eingriffen muss eine vollständige neuromuskuläre Erholung überprüft werden, da bei Patientinnen und Patienten die ohnehin individuelle Eliminationszeit für Muskelrelaxanzien im Zusammenhang mit Organdysfunktionen verlängert sein kann [39].

### Anaphylaktisches Potenzial

Muskelrelaxanzien und Antibiotika besitzen im perioperativen Umfeld mit das höchste Allergiepotenzial [72]. Das Risiko für eine anaphylaktische Reaktion könnte bei Rocuronium etwas höher liegen als bei Atracurium [14, 59]. Atracurium führt im Gegensatz dazu, v. a. bei rascher Injektion, ohne Vorliegen einer Anaphylaxie häufig zu einer dosisabhängigen Histaminliberation. Dies gilt es, bei unklaren hämodynamischen Veränderungen im Rahmen der Einleitung zu bedenken. Bei Patientinnen und Patienten mit hochgradiger Prädisposition für Allergien kann unabhängig vom verwendeten Relaxans die Verabreichung eines Antihistaminikums vor der Narkoseeinleitung erwogen werden. Dadurch können histaminbedingte Reaktionen deutlich abgemildert, immunologisch-vermittelte Hypersensivitätsreaktionen allerdings nicht verhindert werden. Bei dieser Patientengruppe könnte eine Kombination aus Antihistaminikum und Cis-Atracurium das Risiko minimieren [48]. Zur Therapie einer Anaphylaxie auf Rocuronium kann nach Durchführung der Standardtherapie Sugammadex erwogen werden [46, 70].

Auch Sugammadex besitzt – wie jedes Medikament – ein potenzielles Allergierisiko. Erhöhte Inzidenzen an Anaphylaxien, wie in japanischen Studien beschrieben [50, 66], scheinen sich seit der breiten Verwendung nach Wegfall des Patentschutzes bislang nicht gezeigt zu haben, es gilt allerdings, in diesem Zusammenhang wachsam zu bleiben [82] und auch Sugammadex nur bei korrekt gestellter Indikation in der adäquaten Dosierung zu verabreichen [61].

### Leberinsuffizienz

Während Rocuronium überwiegend hepatisch eliminiert wird, wird der Rocuronium-Sugammadex-Komplex renal ausgeschieden. Deshalb scheint die Gabe bei Patientinnen und Patienten mit Leberinsuffizienz bei adäquatem Sicherheitsprofil sinnvoll [25].

### Niereninsuffizienz

Bei einer GFR < 30 ml/min wird die Verwendung von Sugammadex nicht empfohlen. Die Verwendung von Sugammadex in dieser Patientengruppe und sogar bei Patientinnen und Patienten mit terminaler Niereninsuffizienz scheint gemäß aktueller Datenlage sicher zu sein, wobei weitere Studien an großen Patientenzahlen wünschenswert wären [1, 52, 53]. Eine aktuelle Publikation, welche über eine Risikoerhöhung für verschiedene Komplikationen im Vergleich von Rocuronium/Sugammadex gegenüber Cis-Atracurium/Neostigmin bei Vorliegen einer chronischen Niereninsuffizienz berichtet [27], bietet aufgrund der im Artikel beschriebenen methodischen Limitationen aus Sicht des Autors keine adäquate Basis, um daraus aktuell weitere Empfehlungen ableiten zu können. Mangels anderslautender Evidenz sollte diese Information nichtsdestotrotz bekannt sein [16] und die Verwendung von Cis-Atracurium/Neostigmin bei Patientinnen und Patienten mit höhergradiger Niereninsuffizienz erwogen werden.

### Weitere Aspekte

Die Verwendung von Arzneimitteln, die die neuromuskuläre Blockade verstärken können (z. B. Magnesium [28], einige Chemotherapeutika oder Antibiotika wie Aminoglykoside [51]), sollte in der perioperativen Phase nur nach einer Prüfung der Indikationsstellung erfolgen, und die Folgen im Zusammenhang mit der Gabe von Muskelrelaxanzien sollten mittels NMM überwacht werden. Es ist möglich, dass sich beispielsweise der TOF-Quotient und sogar die TOF-Zahl nach Gabe der o. g. Substanzen nach bereits erfolgter (Teil‑)Erholung wieder erniedrigen. Im Gegensatz dazu kann der Betablocker Esmolol den Wirkeintritt eines Muskelrelaxans vermutlich indirekt über die Erniedrigung des Herz-Zeit-Volumens verlängern und im Rahmen einer RSI im ungünstigsten Fall die Intubationsbedingungen verschlechtern [45].

In dieser zusammenfassenden Übersichtsarbeit wird aufgrund des Themenumfangs zu einigen Themen in Infobox 2 auf verschiedene Publikationen mit vertiefenden Inhalten zur jeweiligen Thematik oder Patientengruppe verwiesen.

## Zusammenfassung

Der Themenkomplex Muskelrelaxierung und neuromuskuläres Monitoring ist ein entscheidender anästhesiologischer Baustein der perioperativen Patientensicherheit. Sugammadex ist in diesem Kontext nicht die Lösung aller Probleme, welche mit Muskelrelaxierung einhergehen, sondern ein spezifisches, hochwirksames Medikament, dass bei Kenntnis der pharmakologischen und monitoringspezifischen Details sehr gut eingesetzt werden kann. Allerdings darf dabei keinesfalls, aufgrund einer unbegründeten Sicherheit, die Achtsamkeit verloren gehen. Die Basis zur Vermeidung postoperativer Restblockaden liegt in der Kombination aus adäquater Dosierung, einem korrekt angewandten neuromuskulären Monitoring und der Beurteilung aller weiteren klinischer Informationen – dabei ist physiologisches und pharmakologisches Wissen essenziell.

Zusammenfassend muss das Ziel proklamiert werden, die weiterhin vorherrschenden Unterschiede zwischen den schriftlichen Vorgaben der Guidelines unserer Fachgesellschaften und der klinischen Praxis zu minimieren.

### Infobox 2

- Antagonisierung in der Anästhesie [3]
- Frauen unter hormoneller Kontrazeption Schwangerschaft und Stillzeit [13, 26, 60, 80]
- Kinder [32, 40, 47, 76]
- Geriatrische Patientinnen und Patienten [62]
- Patientinnen und Patienten auf der Intensivstation [39]

## Fazit für die Praxis

- Bei jeder Intubation ist ein Muskelrelaxans zu verwenden.
- Bei jeder Verwendung eines Muskelrelaxans soll ein quantitatives neuromuskuläres Monitoring durchgeführt werden, bevorzugt am N. ulnaris/M. adductor pollicis.
- Vor Beendigung einer Intubationsnarkose sollte ein unter Kenntnis der Limitationen adäquat gemessener TOF-Quotient > 0,95 vorliegen und dokumentiert werden.
- Diverse Substanzen sind zur Erfüllung dieser Ziele verwendbar, wobei sie mit der Kombination aus Rocuronium und Sugammadex häufig am einfachsten erreichbar sind.
- Die Kenntnisse der zugrunde liegenden Physiologie und Pharmakologie bleiben essenziell.

## Data Availability

Alle dieser Arbeit zugrunde liegenden Daten sind in diesem Artikel enthalten.
